# The Clinical Effect of Repetitive Transcranial Magnetic Stimulation on the Disturbance of Consciousness in Patients in a Vegetative State

**DOI:** 10.3389/fnins.2021.647517

**Published:** 2021-04-30

**Authors:** Xiao-Hua Zhang, Ping Han, Yuan-Yuan Zeng, Yu-Long Wang, Hui-Lan Lv

**Affiliations:** ^1^Department of Rehabilitation, Shenzhen Dapeng New District Nanao People’s Hospital, Shenzhen, China; ^2^Department of Rehabilitation, The First Affiliated Hospital, Shenzhen University, Shenzhen Second People’s Hospital, Shenzhen, China

**Keywords:** repetitive transcranial magnetic stimulation, stroke, electroencephalogram, brainstem auditory evoked potential, rehabilitation

## Abstract

**Objective:**

To explore the effect of combining repetitive transcranial magnetic stimulation (rTMS) and conventional rehabilitation on the recovery of consciousness in patients in a persistent vegetative state (PVS).

**Methods:**

A total of 48 patients in a PVS were randomly divided into a treatment and control group. Patients in the treatment group were treated with rTMS to stimulate the dorsolateral prefrontal cortex, and patients in the control group were treated with false stimulation. All patients were evaluated using scales and neuroelectrophysiological assessment before treatment, after 30 days of treatment, and following 60 days of treatment.

**Results:**

Based on the Coma Recovery Scale-Revised (CRS-R) and electroencephalogram (EEG) grading indexes, the treatment group was significantly higher than those of the control group after 30 and 60 days of treatment. The average difference in the three measurements between the two groups before treatment, at 30 days, and 60 days was 0.04, 1.54, and 2.09 for CRS-R and 0.08, −0.83, and −0.62 for EEG indexes, respectively. The latency periods of each wave of the brainstem auditory evoked potentials (BAEPs) in the treatment group were shorter than those in the control group after 30 and 60 days of treatment. In both groups, the BAEP scores after 30 days of treatment were significantly higher than the scores before treatment, and the scores after 60 days of treatment were higher than the scores after 30 days.

**Conclusion:**

In patients in a PVS, rTMS assists in the recovery of consciousness function.

## Introduction

In recent years, owing to rapid developments in emergency medicine and intensive care technology, the mortality rate of critically ill patients has decreased significantly. However, the number of patients with severe consciousness disorders, such as a vegetative state (VS) and minimally conscious state (MCS), has increased dramatically. As a result, arousal research on patients in a VS has attracted significant attention. A VS lasting for more than 1 month is classified as a persistent VS (PVS) (Emergency Medicine Society of Chinese Medical Association, 2002; Xiao and Xu, 2003). Currently, treatments for improving consciousness in patients in a VS include drugs, hyperbaric oxygen treatment, acupuncture, central thalamic deep brain stimulation, and peripheral sensory stimulation programs. These methods have all been reported to affect patients in a VS but each has advantages and disadvantages. Accordingly, finding a safe and effective method for improving the consciousness of patients in a VS remains an urgent problem to be solved.

In recent years, non-invasive neuromodulation technology has received more attention in the fields of neuroscience and rehabilitative medicine (Duan et al., 2015). Repetitive transcranial magnetic stimulation (rTMS) is a physical nerve regulation technology that uses pulsed magnetic fields to act on the central nervous system (primarily the brain) to change the membrane potential of cortical nerve cells, allowing them to generate induced currents, which affects brain metabolism and electrical activity in the nerves, causing a series of physical and biochemical reactions (Cincotta et al., 2015; Mattogno et al., 2017). As a non-invasive and efficient brain function regulation technology, rTMS has shown great potential value in the treatment of chronic pain, neurological diseases, and mental illnesses (Xu et al., 2018). However, few studies have been conducted on the therapeutic effect of rTMS on patients in a VS. The current study investigates the effect of rTMS, combined with conventional rehabilitative therapy, on the recovery of consciousness in patients in a VS, as measured by electroencephalogram (EEG) and brainstem auditory evoked potentials (BAEPs).

## Data and Method

### General Information

This study is a prospective, open, randomized, controlled, single-center clinical trial (clinical trial registration number: ChiCTR2000036073) that was approved by the hospital ethics committee. Forty-eight patients in a PVS were recruited from the rehabilitation department of Nan’ao People’s Hospital in Shenzhen and randomly divided into a treatment and control group (*n* = 24 each). All subjects met the following inclusion criteria: (1) met the diagnostic criteria of a PVS (Ashwal and Cranford, 1995; Coleman, 2002); (2) aged 18–80 years; (3) were in a PVS for the first time; (4) duration of PVS was longer than 3 months; (5) the patient’s family members allowed the patients’ participation in this study, cooperated with doctors’ treatment, and allowed indicators to be collected and measured; (6) a family member or authorized person signed an informed consent form for inclusion in the study. Patients were excluded from the study based on the following exclusion criteria: (1) exhibited a fever, electrolyte disorder, and unstable vital signs; (2) had a late-stage malignant tumor or had undergone radiotherapy or chemotherapy in the preceding 6 months; (3) had implantable electronic devices (e.g., cardiac pacemakers); (4) had intracranial metal implant devices; (5) were pregnant; (6) presented local skin injury or inflammation; (7) exhibited increased intracranial pressure; (8) had serious heart disease; (9) had experienced acute massive cerebral infarction; and (10) had implanted devices containing metal parts in the treatment area.

### Therapeutic Methods

Both patient groups received conventional drug therapy and rehabilitative treatment, including underlying disease treatment and consciousness-regaining drug therapy. The treatment of underlying diseases included antihypertensive treatment, hypoglycemic treatment, antiplatelet agglutination, anticoagulation and statins, and consciousness-regaining therapy including bromocriptine, levodopa, benserazide hydrochloride, and amantadine. Conventional rehabilitative treatment included exercise therapy, traditional rehabilitation therapy, intermediate frequency pulse electrotherapy, and pneumatic therapy.

In the treatment program of the treatment group, the CCY-1 transcranial magnetic stimulator (Yiruide, Wuhan, China) was used in the rTMS mode. According to the recommendation of the International Federation of Clinical Neurophysiology (Rossini et al., 2015), the stimulation intensity was determined based on the resting motor threshold (RMT) of each subject. The RMT is defined as the intensity at which a motor-evoked potential with an amplitude of at least 50 μV can be detected in the abductor pollicis brevis on the affected side at least 5 out of 10 times that a stimulus is applied. The stimulation target was the dorsolateral prefrontal cortex (Heekeren et al., 2006). Patients lay in a supine position during treatment. The surface of the circular coil was at a 45° tangent to the scalp of the affected hemisphere, the stimulation frequency was 5 Hz, and the stimulation intensity was 80% RMT. Each second of stimulation was followed by an interval of 2 s. The total duration of the treatment was 20 min. Therefore, the effective stimulation was 400 strings and 1200 pulses. Treatments were administered once a day, five times a week for 8 weeks.

In the control group, false stimulation was given but the stimulation target, parameters, and duration were the same as those in the treatment group. The operator could hear the magnetic stimulation but the pulsed magnetic field did not enter the brain of the subjects.

### Assessment Methods

#### Clinical Scale Evaluation: The Coma Recovery Scale-Revised

The Coma Recovery Scale-Revised (CRS-R) was modified from the Coma Recovery Scale (2014). The CRS-R comprises six subscales involving hearing, vision, movement, speech, communication, and arousal level. It includes 23 hierarchical and orderly scoring standards. The highest score is 23 points and the higher the score, the milder the disturbance in consciousness (Giacino et al., 2004). For a patient to qualify as being in a VS, the following CRS-R scores are required: auditory ≤ 2; visual ≤ 1; movement ≤ 2; speech ≤ 2; communication = 0; arousal ≤ 2 points. For a patient to be considered as being in an MCS, the following CRS-R scores are required: auditory > 2; visual > 1; movement > 2; speech > 2; communication > 0; arousal > 2 points (Piccione et al., 2011; Chen et al., 2014).

#### Neuroelectrophysiological Assessment Including EEG and BAEPs

All tests were performed by doctors in strict accordance with operating methods. The EEG was performed using a digital EEG instrument (Sigma, United States). Scalp electrodes were placed following the international 10/20 system, and the earlobe was used for the reference electrode. The 20-lead routine tracing mode was adopted. The standard voltage was 100 V/mm, the time constant was 0.3 s, and the high-frequency filter was 30 Hz. The EEG monitoring was performed in a quiet environment and the patient’s eyes were closed. The monitoring duration was 20 min. After monitoring, the EEG frequency and amplitude were calculated. According to Hockaday’s (1965) EEG grading standard for consciousness disorders, the EEGs of comatose patients were analyzed. Grade I (normal range): (1) α rhythm; (2) α rhythm was dominant, accompanied by a few θ waves. Grade II (mild abnormality): most of the waves were θ waves, accompanied by a few δ waves. Grade III (moderate abnormality): (1) δ waves, mixed with θ waves and a few α waves; (2) primarily δ waves and no other rhythmic activities. Grade IV (serious abnormality): (1) diffuse δ waves accompanied by short-term electrical rest; (2) some leads with scattered δ waves while other leads showed electric rest. Grade V (extreme abnormality): (1) an almost flat wave; (2) no EEG activity (Chen et al., 2011).

The BAEPs were examined using an electromyographically evoked potential meter (NEUROWERK EMG, SIGMA Medizin-Technik, Germany). In the BAEP procedures, short sounds (clicks) were used to stimulate both ears separately. The unstimulated ear was masked with white noise. Both sides were traced simultaneously. The stimulus intensity was 75 dB, superimposed 2000 times. The analysis time was 10 ms and testing was repeated at least twice for each ear. The coincidence of each wave, the peak latency of grade I, III, and V waves, and the latency between the peaks of grade I and grade III waves and between grade III and V waves were recorded. In some instances, grade II and IV waves were missing and no recording was made.

#### Evaluation Timeframe

The CRS-R, EEG, and BAEP were evaluated once each before treatment, after 30 days of treatment, and after 60 days of treatment.

### Statistical Analysis

Data were analyzed using the SPSS Statistics 22.0 statistical software. Count data were evaluated using a chi-square test. Measurement data were expressed as mean ± standard deviation (x ± SD), and data before and after treatment within each group were compared using a paired sample *t* test. The differences before and after treatment were compared between the two groups using univariate analysis of variance (ANOVA). After establishing the regression equation, repeat measurement ANOVA was performed; *P* < 0.05 was considered statistically significant.

## Results

During rehabilitative treatment, there were no adverse reactions associated with epileptic seizures in either of the groups. The 48 people who had been selected as the subjects of the study were randomly divided into a treatment and a control group (*n* = 24 in both groups). In the control group, there were 17 males (70.8%) and 7 females (29.2%). Their average age was 53.83 ± 12.38 years. Similarly, in the treatment group, there were 17 males and 7 females. Their average age was 56.13 ± 14.16 years. The results of the chi-square test and the independent sample *t* test revealed no significant differences between the control and treatment groups in terms of gender and average age, i.e., the subjects’ background data were consistent (Table 1).

**TABLE 1:** Comparison of subjects’ data.

	Gender		Age (years old)
	Male	Female	
Control group	17 (70.8%)	7 (29.2%)	53.83 ± 12.38
Treatment group	17 (70.8%)	7 (29.2%)	56.13 ± 14.16
χ^2^/*t*	0.000		0.597
*p*	1.000		0.553

**FIGURE 1:**
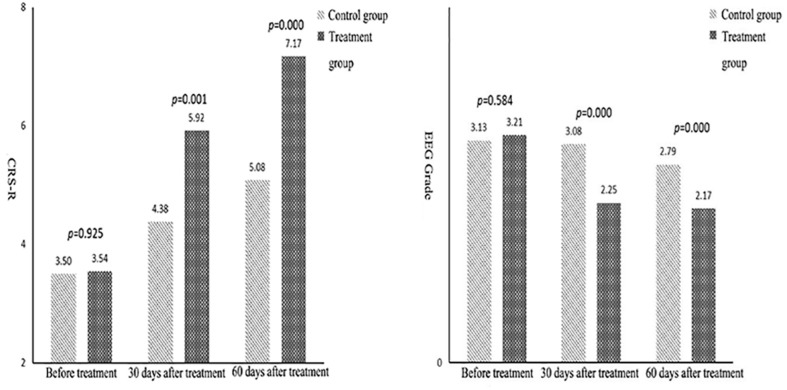
Comparison of mean Coma Recovery Scale-Revised and electroencephalogram grades between the two groups.

**FIGURE 2:**
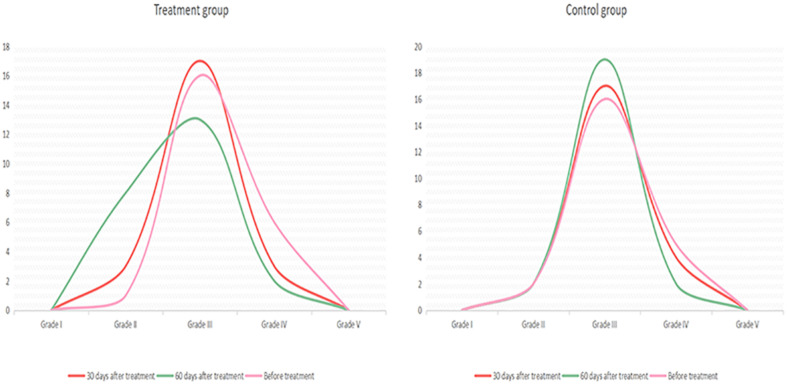
Electroencephalogram changes in the treatment and control groups before and after treatment.

An independent sample *t* test was used to compare the mean scores of the CRS-R and EEG indicators of the control and treatment groups at each time point. The repeated ANOVA measurement was used to compare the results of the three measurements within the groups. A pairwise comparison was then carried out to assess the statistical significance of the differences in the results. The specific test results are shown in Tables 2, 3, and Figures 1, 2.

**TABLE 2:** Difference test of index scores before treatment, at 30 days after treatment, and 60 days after treatment.

Groups	Time point	CRS-R	EEG
Control group	Before treatment	3.50 ± 1.47	3.13 ± 0.54
	30 days after treatment	4.38 ± 1.31^②^	3.08 ± 0.50^②^
	60 days after treatment	5.08 ± 1.79^②③^	2.79 ± 0.51^②③^
Treatment group	Before treatment	3.54 ± 1.56	3.21 ± 0.51
	30 days after treatment	5.92 ± 1.59^①②^	2.25 ± 0.44^①②^
	60 days after treatment	7.17 ± 2.04^①②③^	2.17 ± 0.38^①②③^

*① means that the mean values of the treatment group and the control group were significantly different at the same time point. ② means that the mean value at this time point is significantly different from that before treatment. ③ means that the mean value at this time point is significantly different from that after 30 days of treatment.*

**TABLE 3:** EEG changes of the treatment group and the control group before and after treatment.

Group		Treatment group	Control group
Before treatment	Grade I	0	0
	Grade II	1	2
	Grade III	16	16
	Grade IV	6	5
	Grade V	0	0
30 days after treatment	Grade I	0	0
	Grade II	3	2
	Grade III	17	17
	Grade IV	3	4
	Grade V	0	0
60 days after treatment	Grade I	0	0
	Grade II	8	2
	Grade III	13	19
	Grade IV	2	2
	Grade V	0	0

The data in Table 2 reveal that, in terms of the EEG grade indexes, the mean difference between the control and treatment groups was not significant, but the score of the treatment group was significantly higher than in the control group after 30 and 60 days of treatment. The average difference in measurements before treatment, after 30 days, and after 60 days of treatment was 0.08, −0.83, and −0.62, respectively. This means that over time, the difference between the results in the control and the treatment groups tended to increase. That is, the scores after 30 days of treatment were significantly higher than those before treatment, and the scores after 60 days of treatment were higher than those after 30 days.

An independent sample *t* test was used to compare the mean scores of grades I, III, and V waves, and grades I–III and grades III–V wave intervals between the control and treatment groups at each point in time. A repeated ANOVA measurement was then used to compare the results of the three measurements within each group. A pairwise comparison was then carried out to assess the statistical significance of the differences in the results between the two groups. The specific test results are shown in Table 4.

**TABLE 4:** Difference test of BAEP latency score between the two groups after treatment.

Group	Time point	Grade I waves	Grade III waves	Grade V waves	The peaks of the grade I and the grade III wave	The peaks of the grade III and the grade V wave
Control group	Before treatment	1.91 ± 0.12	4.25 ± 0.17	6.94 ± 0.25	2.30 ± 0.10	2.32 ± 0.23
	30 days after treatment	1.91 ± 0.12 ^②^	4.21 ± 0.16^②^	6.83 ± 0.24^②^	2.19 ± 0.10^②^	2.21 ± 0.23^②^
	60 days after treatment	1.90 ± 0.12^②③^	4.15 ± 0.16^②③^	6.82 ± 0.23^②③^	2.16 ± 0.08^②③^	2.18 ± 0.20^②③^
Treatment group	Before treatment	1.90 ± 0.13^①^	4.23 ± 0.18^①^	6.95 ± 0.27^①^	2.31 ± 0.12^①^	2.32 ± 0.23^①^
	30 days after treatment	1.81 ± 0.15^①②^	4.03 ± 0.14^①②^	6.25 ± 0.29^①②^	2.10 ± 0.13^①②^	2.08 ± 0.16^①②^
	60 days after treatment	1.69 ± 0.09^①②③^	3.91 ± 0.10^①②③^	5.78 ± 0.41^①②③^	2.02 ± 0.05^①②③^	1.91 ± 0.09^①②③^

*① means that the mean values of the treatment group and the control group were significantly different at the same time point. ② means that the mean value at this time point is significantly different from that before treatment. ③ means that the mean value at this time point is significantly different from that after 30 days of treatment.*

**FIGURE 3:**
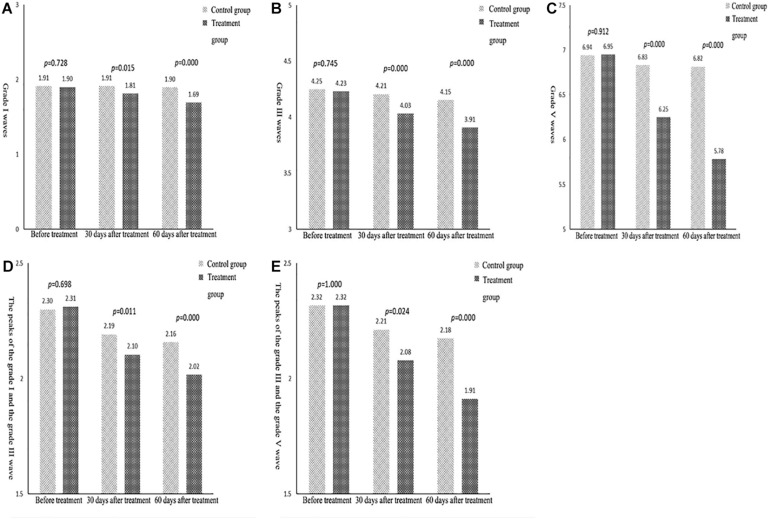
**(A)** Comparison of the mean indexes of grade I waves between the two groups. **(B)** Comparison of the mean indexes of grade III waves between the two groups. **(C)** Comparison of the mean indexes of grade V waves between the two groups. **(D)** Comparison of the mean indexes of grade I–III waves between the two groups. **(E)** Comparison of the mean indexes of grade III–V waves between the two groups.

In terms of the indexes for the grade I wave, the difference between the control and the treatment groups before treatment was not statistically significant. The results after 30 and 60 days of treatment revealed significantly higher scores for the treatment group compared with the control group. The average difference in measurements before treatment, after 30 days, and after 60 days of treatment was 0.01, 0.10, and 0.20, respectively. This indicated that, over time, the difference between the results in the control and the treatment groups tended to increase; that is, the scores after 30 days of treatment were significantly higher than those before treatment, and the scores after 60 days of treatment were higher than those after 30 days (Figure 3A). In terms of the indexes for the grade III wave, the differences between the control and treatment groups before treatment were not statistically significant. The measurement results after 30 and 60 days of treatment revealed that the scores of the treatment group were significantly higher than those of the control group; the average difference in the three measurements was 0.02, 0.18, and 0.25; that is, over time, the difference between the two tended to increase. In the three measurement results for the same group, the scores after 30 days of treatment were significantly higher than those before treatment, and the scores after 60 days of treatment were both higher than those after 30 days and before treatment (Figure 3B).

Similarly, for the indexes of the grade V wave (Figure 3C), grades I–III wave intervals (Figure 3D), and grades III–V wave intervals (Figure 3E), the results showed no statistically significant difference between the treatment and the control group before treatment. The results also showed that the difference between the two groups tended to increase over time. The scores after 30 days of treatment were significantly higher than those before treatment, and the scores after 60 days were higher than those after 30 days.

## Discussion

In this randomized, pseudo-controlled study, 48 patients in a PVS were treated with high-frequency rTMS or false rTMS stimulation of (DLPC) for 60 days. Additionally, CRS-R, EEG, and BAEP were assessed before treatment, after 30 days, and after 60 days of treatment. The CRS-R scale was used to diagnose patients in a VS or an MCS. When all six items on the CRS-R scale were scored, the patients could be diagnosed as either being in a VS or an MCS. The CRS-R scores required for the diagnosis of a VS are hearing ≤ 2; vision ≤ 1; movement ≤ 2; speech response ≤ 2; communication = 0; arousal ≤ 2 points. The CRS-R scores required for the diagnosis of an MCS are hearing > 2; vision > 1; movement > 2; speech response > 2; communication > 0; arousal > 2 points. The results revealed that after 30 days of treatment, the CRS-R scores of two of the patients in the treatment group had increased but no obvious change was found in clinical behavior. They had not moved into an MCS or a waking state. In the control group, no significant change in the CRS-R score was found after 30 days of treatment. After 60 days of treatment, the CRS-R score of 12 patients in the treatment group had increased. Five of the patients were in an MCS. In the control group, the scores of five patients were higher than before; one of the patients was in an MCS and the remaining four were still in a VS.

The analysis of statistical results after 30 and 60 days of treatment revealed that the scores of the treatment group were significantly higher than those of the control group. The average difference between the measurements before treatment, after 30 days, and after 60 days of treatment was 0.04, 1.54, and 2.09, respectively. This indicated that the difference between the two groups tended to increase over time. The three results within each group showed that the scores after 30 days of treatment were significantly higher than those before treatment, and the scores after 60 days of treatment were higher than those after 30 days of treatment. This showed that an improvement in consciousness was greater in the treatment group than in the control group, i.e., the therapeutic effect was greater in the rTMS group than in the false rTMS stimulation group.

An electrophysiological examination can objectively evaluate disturbances in consciousness and the prognosis of patients. In such evaluations, EEG and BAEP are commonly used (Cincotta et al., 2015). In this study, quantitative analysis using EEG data proved more sensitive for detecting delicate cortical activity (Al-Qazzaz et al., 2014). Based on the results herein, before treatment, the EEG characteristics of the two groups were grade I and grade V in 0 patients, grade III in most patients, and grade IV in a few patients. In the treatment group, after 30 days of treatment, three patients had recovered from grade IV to grade III, and two patients had recovered from grade III to grade II. After 60 days of treatment, one patient had recovered from grade IV to grade III and six patients had recovered from grade III to grade II. In the control group, after 30 days of treatment, one patient had recovered from grade IV to grade III. After 60 days of treatment, two patients had recovered from grade IV to grade III. These results revealed that, aside from two patients, the patients in the treatment group all had an improved CRS-R score. Although five patients showed no significant change in CRS-R score, the slow wave decreased in the EEG waveform, α rhythm appeared, or α-wave amplitude increased more obviously, and the overall EEG grade improved. This revealed that changes in EEG results could be observed before perceiving changes in clinical behavior. In the treatment group, after 60 days of treatment, in the five patients whose diagnosis changed from a VS to an MCS, the EEG results also improved significantly. In the treatment group, the overall improvement in EEG results was more pronounced than in the control group. The CRS-R scale is more subjective and less sensitive;(Rossini et al., 2015); therefore, the functional status of patients diagnosed to be in a VS can be evaluated more fully using EEG assessment because EEG response is more sensitive than CRS-R and, as such, can be considered an early indicator of consciousness recovery (Neto et al., 2015).

BAEP reflects the integrity of the brainstem auditory pathway and the functioning of the brainstem and the auditory nerve. A study found that a shorter latency indicated the improvement of hearing pathway conduction disorder (Soustiel et al., 1993). This can be used as an indicator for prognosis and to evaluate the curative effect in patients with a disturbance in consciousness after brain injury (Zhang, 2016; Zhao et al., 2018). In this study, in all subjects, various waveforms and interwave latencies could be induced. A comparison was conducted between the two groups at all points in time, i.e., between the treatment group and the control group before treatment, after 30 days of treatment, and after 60 days of treatment. The data in Table 4 reveal that there were no significant differences between the control and the treatment groups in terms of the latency period for BAEPs grade I, III, V, I–III, and III–V before treatment. The results after 30 and 60 days of treatment revealed that the latency periods of each BAEP wave in all grades in the treatment group were shorter than those in the control group (*P* < 0.05).

There was a significant statistical difference between the treatment and control groups, which indicated that the treatment effect in the treatment group was better than in the control group. When examining the results within the groups, the scores after 30 days of treatment were significantly higher than those before treatment, and the scores after 60 days of treatment were higher than those after 30 days. The results revealed that high-frequency rTMS treatment can effectively improve the electrophysiological activity of patients in a PVS and promote the recovery of consciousness.

Conversely, an independent sample *t* test was used to compare the mean scores of the CRS-R and EEG grading indexes. The differences between the control and treatment groups before treatment were not statistically significant; however, the results after 30 and 60 days of treatment revealed that the scores in the treatment group were significantly higher than those in the control group. The average difference between the three measurements was 0.08, −0.83, and −0.62, respectively. This indicated that the difference between the two groups tended to increase over time. Within each group, the scores after 30 days of treatment were significantly higher than those before treatment, and the scores after 60 days of treatment were higher than those after 30 days. The difference in the latency of BAEPs grade I, III, V, I–III, and III–V increased over time between the two groups. The results revealed that rTMS may have a long-lasting TMS effect. Some changes can only be observed after a long period of rTMS treatment and the EEG power is increased. We speculate that this may be related to brain plasticity.

In summary, in this study, the clinical CRS-R scale was used to evaluate the effect of rTMS on patients in a VS. An EEG was used to monitor the function of the brain at the level of cortical information processing and to observe any changes in unconscious and conscious states. BAEP was used to evaluate the integrity of the brainstem auditory pathway, as well as the function of the brainstem and the auditory nerve. The combination of EEG and BAEP dynamic detection can provide more comprehensive and accurate information for the evaluation of the curative effect and the prognosis of patients in a PVS. The study results revealed that rTMS can promote the recovery of consciousness in patients in a VS and can be used as a rehabilitative treatment for patients with a disturbance in consciousness.

Additionally, rTMS was applied through a pulsed magnetic field to generate an inductive current acting on the brain tissue. The nerve cells are depolarized and evoked potentials are produced to activate the cortex and to change the physiological process in the brain as a means for deriving the localization of cortical function. In addition, by changing the excitability of the local cortex, the cortical metabolism and brain blood flow can be affected, and brain tissue plasticity can be regulated, thereby promoting brain function recovery. Salma et al. (Schutter, 2011) stimulated the dorsolateral prefrontal cortex using rTMS at 10 Hz. Through transcranial Doppler (TCD), the cerebral blood flow rate of patients with cerebral infarction was accelerated and brain function was improved. Rossi et al. (Sun et al., 2011) showed that transcranial magnetic stimulation can change the ion channel of cell membranes, change cell excitability, induce lateral branch budding of axons, and promote nerve cell regeneration to repair the central nervous system.

Additionally, rTMS also has specific effects on neurotransmitters and various receptors in the brain, which, in turn, may serve as mechanisms to improve rTMS improve brain function. As early as 1998, Ziemann et al. (1998) found that high-frequency rTMS can promote the release of excitatory neurotransmitters, such as dopamine, glutamate, and other excitatory neurotransmitter content, which increases nerve conduction velocity and, as such, plays a role in stimulating the brain, which is important for maintaining awareness.

Li et al. (Yue et al., 2009) also found that after 15 days of stimulation with low-frequency (0.5 Hz) rTMS, the content of glutamic acid and aminobutyric acid in the hippocampus and striatum of rats increased significantly. The anatomical basis of arousal in this instance is the uplink reticular activation system, a network of neurons radiating from the pons to the mesencephalon through the thalamus to the bilateral hemispheres. The sensory conduction pathway continues up to the brainstem reticular structure, and its uplink impulse is projected to a wide area of the cerebral cortex through non-specific projection after thalamic transformation, leaving the cerebral cortex in an excited state that can maintain arousal. In this study, after treatment, the consciousness of patients in a plant state improved. As such, the mechanism of action may be able to effect an increase in dopamine and norepinephrine tryptamine content in the hindbrain, thereby activating the uplink reticular structure of the brainstem, placing the cerebral cortex in an excited state, and promoting the recovery of consciousness.

Additional studies (Kleinjung et al., 2008) have shown that directly applying rTMS to the local cerebral cortex, which is stimulated locally, and indirectly acts on the distant part related to its function through a nerve fiber connection, thus changing the functional state of the distant part cortex.

However, the nature of the relationship between the efficacy of treatment and the site of magnetic stimulation, and the frequency and intensity of stimulation requires additional investigation. A comparison of the effects of different stimulation frequencies and intensities is required to achieve the best possible therapeutic effect. The long-term sequelae effect of rTMS is a study topic that our research team will explore next.

## Data Availability Statement

The original contributions presented in the study are included in the article/supplementary material, further inquiries can be directed to the corresponding authors.

## Ethics Statement

The studies involving human participants were reviewed and approved by the First Affiliated Hospital, Shenzhen University, Shenzhen Second People’s Hospital. The patients/participants provided their written informed consent to participate in this study. Written informed consent was obtained from the individual(s) for the publication of any potentially identifiable images or data included in this article.

## Author Contributions

X-HZ and Y-LW conceived the idea, conceptualized the study, and drafted the manuscript. PH collected the data. Y-YZ analyzed the data. H-LL reviewed the manuscript. All authors read and approved the final draft.

## Conflict of Interest

The authors declare that the research was conducted in the absence of any commercial or financial relationships that could be construed as a potential conflict of interest.
